# Association between the depression and cardiovascular risk in arthritis patients: a prospective cohort study from the CHARLS database

**DOI:** 10.3389/fimmu.2025.1590483

**Published:** 2025-08-19

**Authors:** Xiaoyuan Tian, Xianglin Yang, Ying Cao, Zhenan Qu, Bocheng Zhang

**Affiliations:** ^1^ Department of Orthopaedics, Second Affiliated Hospital, Dalian Medical University, Dalian, China; ^2^ Department of Orthopaedics, Affiliated Zhongshan Hospital, Dalian University, Liaoning, Dalian, China

**Keywords:** arthritis, depression, CHARLS, cardiovascular disease, inflammatory status

## Abstract

**Objective:**

To investigate the association between depression and cardiovascular disease (CVD) risk in arthritis patients and evaluate the modifying role of systemic inflammation.

**Methods:**

Using data from the China Health and Retirement Longitudinal Study (CHARLS), we conducted a prospective cohort analysis of 2,571 arthritis patients without baseline CVD. Depression severity was assessed using the 10-item CES-D scale, with scores ≥12 defining depression. Multivariable logistic regression and restricted cubic spline (RCS) models analyzed dose-response relationships. We utilized C-reactive protein (CRP) to assess whether inflammatory status in arthritis patients modify this relationship. Sensitivity analyses included multiple imputation and complete-case approaches.

**Results:**

Each 1-point increase in CES-D score elevated CVD risk by 2.8% (OR=1.028, 95% CI 1.012–1.044). High inflammation amplified CVD risk exclusively in depressed patients (OR=1.52 vs. OR=1.32 in low-inflammation depressed group), with no significant association in non-depressed individuals. Diabetes mellitus synergistically intensified this relationship (OR=2.83 in diabetic vs. OR=1.21 in non-diabetic depressed patients, P = 0.03 for interaction). Results remained robust across sensitivity analyses.

**Conclusion:**

Depression linearly increases CVD risk in arthritis patients, with systemic inflammation selectively potentiating this association in depressed individuals. The diabetes-depression-CVD interaction highlights shared pathophysiological pathways. These findings underscore the imperative for integrated clinical strategies targeting both psychological health and inflammatory pathways to reduce cardiovascular morbidity in arthritis populations.

## 1 Introduction

Arthritis, a group of conditions characterized by joint pain, inflammation, and stiffness, is a leading cause of disability worldwide, with osteoarthritis (OA) and rheumatoid arthritis (RA) being the most prevalent (1–3). Globally, arthritis represents a major public health challenge, significantly affecting both the quality of life and functional independence of millions of individuals, particularly in older adults. Both OA and RA carry significant health burdens beyond just joint discomfort. Beyond their direct musculoskeletal burden, arthritis patients exhibit a 1.5 to 2 fold elevated risk of cardiovascular disease (CVD) compared to age- and sex-matched controls (4). While chronic inflammation-driven endothelial dysfunction and accelerated atherosclerosis are well-recognized mechanisms (5), emerging evidence suggests that psychological comorbidities, particularly depression, may exacerbate this risk through bidirectional neuroimmune pathways (6–8).

Depression is also a common comorbidity of arthritis, particularly in RA. Studies have shown that 14% to 48% of patients with RA experience varying degrees of depression (9, 10), which are 2 to 3 times higher than in the general population (11, 12). A meta-analysis that included 49 studies indicated that the pooled prevalence rate of depression among OA patients was 19.9% (95% CI 15.9–24.5%), which was significantly higher than that in age-matched non-OA populations (RR = 1.17, 95% CI 0.69–2.00). Depression reduces the likelihood of achieving disease remission and treatment response in arthritis patients (13), while concurrently exacerbating pain, fatigue, and physical disability (14). Furthermore, it significantly increases healthcare costs and diminishes health-related quality of life (15, 16). Depression is not merely a reactive psychological state but a systemic inflammatory condition that amplifies cytokine-mediated vascular damage (e.g., IL-6, TNF-α) and dysregulates autonomic nervous system activity (17). Previous studies have demonstrated a significant association between depression and both CVD and cardiovascular-related mortality (18). A meta-analysis incorporating 26 studies revealed that depression is associated with a 16% increased risk of cardiovascular events (HR = 1.16, 95% CI 1.04-1.30) and a 44% higher cardiovascular-related mortality rate (HR = 1.44, 95% CI 1.27-1.60) (19). While depression is recognized as a contributor to CVD in the general population, its role in arthritis-specific cohorts—particularly in the context of chronic inflammation—remains poorly characterized.

Unlike arthritis, depression is treatable. Given the association between depression and increased CVD risk in arthritis patients, early identification and intervention for depression are crucial. Therefore, there is an urgent need to investigate the relationship between depression and incident CVD. Furthermore, the potential role of inflammatory activity in mediating this association remains to be elucidated. However, there is a lack of evidence on the relationship. To address these knowledge gaps, we utilized data from the prospective cohort of the China Health and Retirement Longitudinal Study (CHARLS). Our primary aim was to examine the association between depression and CVD in individuals with arthritis. Additionally, we utilized C-reactive protein (CRP) to assess whether inflammatory status in arthritis patients modify this relationship. Our findings may inform integrated care models targeting both mental health and inflammation to reduce CVD burden in arthritis patients.

## 2 Methods

### 2.1 Study population and design

The CHARLS is a nationally representative longitudinal survey of persons in China 45 years of age or older and their spouses, including assessments of social, economic, and health circumstances of community-residents (20). The current study uses data from the first wave (2011) of CHARLS as the baseline and data from the fourth wave (2018) as the study endpoint, as these waves included self-reported CVD from the participants. The entire study process adhered to the principles outlined in the Declaration of Helsinki, and the study results were reported in accordance with the Strengthening the Reporting of Observational Studies in Epidemiology (STROBE) guidelines (21). The protocol for the CHARLS cohort was approved by Peking University’s Ethics Review Committee (IRB00001052–11015), and all participants provided written informed consent prior to their participation.

**Figure 1:**
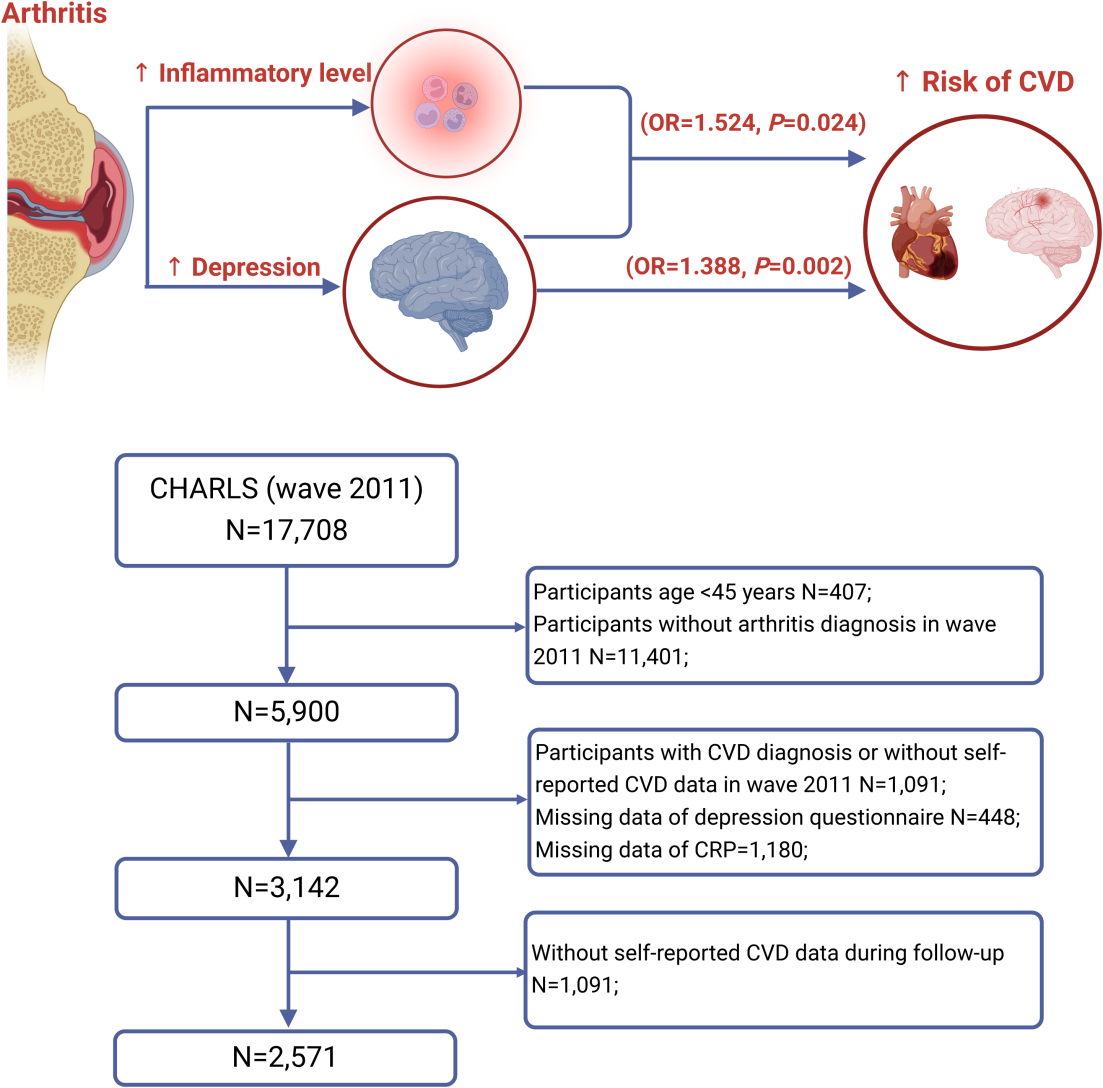
Flow chart of the study. CHARLS, China Health and Retirement Longitudinal Study; CVD, cardiovascular disease; CRP, C-reactive protein.

**Figure 1** shows the screening process for the study population in the current study. Among the 17,708 participants from the first wave, we excluded those participants aged 45 years and below (n = 407), those without arthritis diagnosis at 2011 (n = 11,401). We excluded participants who were diagnosed with CVD in 2011 and those with missing CVD data (n = 1,091). Additionally, we excluded participants who did not complete the depression questionnaire (n = 448) and those with missing data on CRP (n= 1,180). Finally, participants without self-reported CVD information during follow-up (n = 571) were also excluded. Ultimately, 2,571 participants were included in the current analysis.

### 2.2 Diagnosis of cardiovascular disease

The diagnosis of CVD was based on self-reported heart disease or stroke, including heart disease and stroke. Similar to previous studies (22), the interviewer will ask the participants a question, such as “Have you been told by a doctor that you have been diagnosed with a heart attack, coronary heart disease, angina, congestive heart failure, or other heart problems?” or “Have you been told by a doctor that you have been diagnosed with a stroke?” Participants who reported heart disease or stroke were defined as having CVD. Participants who had diagnosed CVD at 2011 were excluded, and if the participants were diagnosed with CVD until the follow-up period in 2018, they were included in the study and were defined as having new-onset CVD.

### 2.3 Diagnosis of depression

The severity of depression is evaluated utilizing the CES-D (23), comprising 10 items that ascertain the frequency of depressive symptoms during the preceding one week. Each item is associated with four potential answers, denoted as “ Rarely or none of the time”, “ Some or a little of the time”, “ Occasionally or a moderate amount of the time” and “ Most or all of the time”, corresponding to scores ranging from 0 to 3. The CES-D total score varies from 0 to 30, with a higher score indicating more depressive symptoms (24). Previous research has demonstrated that a threshold score of 12 represents the standard cut-off score for achieving the highest specificity and sensitivity in diagnosing depression among Chinese older adults (25). Consequently, our study defined individuals with a score equal to or greater than 12 as indicative of depression.

### 2.4 Definition of covariates

The covariates primarily encompassed those linked to depression or cardiovascular risk, which were ascertained via a comprehensive literature review or expert consultation. Covariates mainly include demographic data, lifestyle and metabolic factors. We included age, sex, marital status and residence place to adjust for differences in demographic data. The residence place was categorized as rural or urban. Lifestyle covariate included the drinking and smoking state. Metabolic risk factors include hypertension, diabetes mellitus (DM), body mass index (BMI) and waist circumference (WC). Hypertension was defined as systolic blood pressure (SBP) ≥ 140 mmHg, diastolic blood pressure (DBP) ≥ 90 mmHg or being told by a doctor that they had hypertension. DM was defined as fasting glucose ≥ 7.0 mmol/L, glycated hemoglobin (HbA1c) ≥ 6.5%, random blood glucose ≥ 11.1 mmol/L or being told by a doctor that they had DM. We also classified blood lipids laboratory test results as confounding factors because they have a strong correlation with CVD. Laboratory test results included total cholesterol (TC), triglycerides (TG), low-density lipoprotein cholesterol (LDL-C), high-density lipoprotein cholesterol (HDL-C).

### 2.5 Statistical analyses

All statistical analyses were conducted by R software (version 4.4.1). Means and standard errors (SE) were used to present continuous variables, while percentages were used for categorical variables. Baseline characteristics were compared using T-tests for continuous variables and chi-square tests for categorical variables. We have established three multivariable logistic regression models between CES-D score (continuous variable) and CVD: Crude model, no covariates were adjusted; Model 1: age, sex, marital status, education level and residence place were adjusted; Model 2: age, sex, marital status, education level, residence place, smoking, drinking state, BMI, WC, hypertension, DM and blood lipids laboratory test results were adjusted. Results from the logistic regression analysis are reported as odds ratios (ORs) with 95% confidence intervals (CIs). We assessed potential multicollinearity among variables in each model using the variance inflation factor (VIF). The VIF values for all variables in each model were below 10 and no significant multicollinearity problems were detected (**Supplementary Table 1**). A restricted cubic spline (RCS) model with three equally spaced nodes was used to investigate the nonlinear and dose-response trends association between CES-D score and the risk of CVD. Subsequently, we ascertained participants’ depressive status using a CES-D score of 12 as the cut-off score and examined the association between depression status (categorical variable) and CVD.

To evaluate the potential role of inflammatory activity in the association between depression and cardiovascular events among arthritis patients, we classified participants into two inflammatory strata based on CRP levels: low-grade inflammation (CRP <3 mg/L) and high-grade inflammation (CRP ≥3 mg/L) (26). Subsequently, participants were categorized into four mutually exclusive groups according to their depression status (presence/absence) (1): Low inflammation without depression; (2) Low inflammation with comorbid depression; (3) High inflammation without depression; (4) High inflammation with comorbid depression. Stratified analyses were performed based on gender (male or female), age (< 60 or ≥ 60 years old), residence place (rural or urban), BMI (normal weight: BMI < 24.0, overweight: 24.0 ≤ BMI < 28.0 or obesity: BMI ≥ 28.0), smoking state (yes or no), and drinking state (yes or no) and comorbidities to explore their potential modifying effects, which were assessed by testing multiplicative interaction terms. “Mediation” package was utilized to perform Mediation analysis assessing the mediating effects of depression and CRP with the cardiovascular risk, adjusted by age, sex, marital status, education level, residence place, smoking, drinking state, BMI, WC, hypertension, DM and blood lipids laboratory test results (27, 28). Three sensitivity analyses were conducted as follows: (1) using multiple imputation to address missing data for covariates. The multiple imputation by chained equations method was utilized to impute the missing data through the “MICE” package of the R software (29); (2) excluding participants with missing data on covariates. P value < 0.05 is considered to have statistical difference.

## 3 Result

### 3.1 Baseline characteristics

A total of 2,571 individuals met the inclusion criteria in this study. The average age was 58.72 years old, 59% were female, and 70.4% were rural. The mean CES-D score was 9.96, with 1,226 participants (47.69%) scoring above 12, which was defined as indicative of depression. There were significant differences between the two groups in age, sex, marital status, education level, residence place, smoking, drinking state, BMI, WC and HDL levels. During the follow-up period, 579 individuals were diagnosed with new-onset CVD (heart disease, 432 cases; stroke, 203 cases), and the incidence of CVD was 22.52%. Baseline characteristics are presented in **Table 1**. To assess potential selection bias, we compared the baseline characteristics of the excluded participants with those retained in the study (**Supplementary Table 2**).

**Table 1:** Baseline characteristics of the study population.

Variable	Total (n=2571)	Arthritis without depression (n=1345)	Arthritis with depression (n=1226)	p.value
Age (yr), Mean (SE)	58.72 ± 8.51	58.27 ± 8.48	59.20 ± 8.52	**<0.01**
Sex				**<0.0001**
Female n (%)	1515 (59.00)	695 (51.75)	820 (66.94)	
Male n (%)	1053 (41.00)	648 (48.25)	405 (33.06)	
Marital status				**<0.0001**
Married n (%)	2292 (89.15)	1238 (92.04)	1054 (85.97)	
Other n (%)	279 (10.85)	107 (7.96)	172 (14.03)	
Residence				**<0.0001**
Rural n (%)	1810 (70.40)	884 (65.72)	926 (75.53)	
Urban n (%)	761 (29.60)	461 (34.28)	300 (24.47)	
Educational level				**<0.0001**
Middle school or above n (%)	614 (23.90)	411 (30.58)	203 (16.57)	
No formal education n (%)	1358 (52.86)	611 (45.46)	747 (60.98)	
Primary school n (%)	597 (23.24)	322 (23.96)	275 (22.45)	
CES-D score, Mean (SE)	9.96 ± 6.54	4.83 ± 2.71	15.59 ± 4.57	**<0.0001**
BMI (kg/m2), Mean (SE)	23.56 ± 3.94	23.94 ± 4.04	23.13 ± 3.79	**<0.0001**
WC (cm), Mean (SE)	83.90 ± 12.64	84.73 ± 13.09	82.97 ± 12.07	**<0.001**
CRP (mg.L), Mean (SE)	2.48 ± 5.92	2.44 ± 5.56	2.53 ± 6.29	0.7
TC (mg.dL), Mean (SE)	195.45 ± 39.55	195.46 ± 40.92	195.43 ± 38.00	0.98
HDL-C (mg.dL), Mean (SE)	52.15 ± 15.45	51.08 ± 15.06	53.33 ± 15.79	**<0.001**
LDL-C (mg.dL), Mean (SE)	116.65 ± 35.04	116.51 ± 34.91	116.79 ± 35.19	0.84
TG (mg.dL), Mean (SE)	137.11 ± 124.00	141.03 ± 130.51	132.79 ± 116.32	0.09
Smoking status				**<0.0001**
No n (%)	1867 (72.62)	914 (67.96)	953 (77.73)	
Yes n (%)	704 (27.38)	431 (32.04)	273 (22.27)	
Alcohol status				**<0.01**
No n (%)	1738 (67.60)	871 (64.76)	867 (70.72)	
Yes n (%)	833 (32.40)	474 (35.24)	359 (29.28)	
DM				0.67
No n (%)	2242 (87.20)	1177 (87.51)	1065 (86.87)	
Yes n (%)	329 (12.80)	168 (12.49)	161 (13.13)	
Hypertension				0.86
No n (%)	1652 (64.28)	862 (64.09)	790 (64.49)	
Yes n (%)	918 (35.72)	483 (35.91)	435 (35.51)	
CVD				0.05
No n (%)	1992 (77.48)	1063 (79.03)	929 (75.77)	
Yes n (%)	579 (22.52)	282 (20.97)	297 (24.23)	

yr, year; SE, standard error; BMI, body mass index; WC, waist circumference; CRP, C-reactive protein; TC, total cholesterol; HDL-C, high-density lipoprotein cholesterol; LDL-C, low-density lipoprotein cholesterol; TG, triglycerides; DM, diabetes mellitus; CVD, cardiovascular disease; All the above mean, standard error, and percentage of frequency were weighted except for the frequency.

Significant values are in bold.

### 3.2 Depression and CVD in arthritis

**Figure 2:**
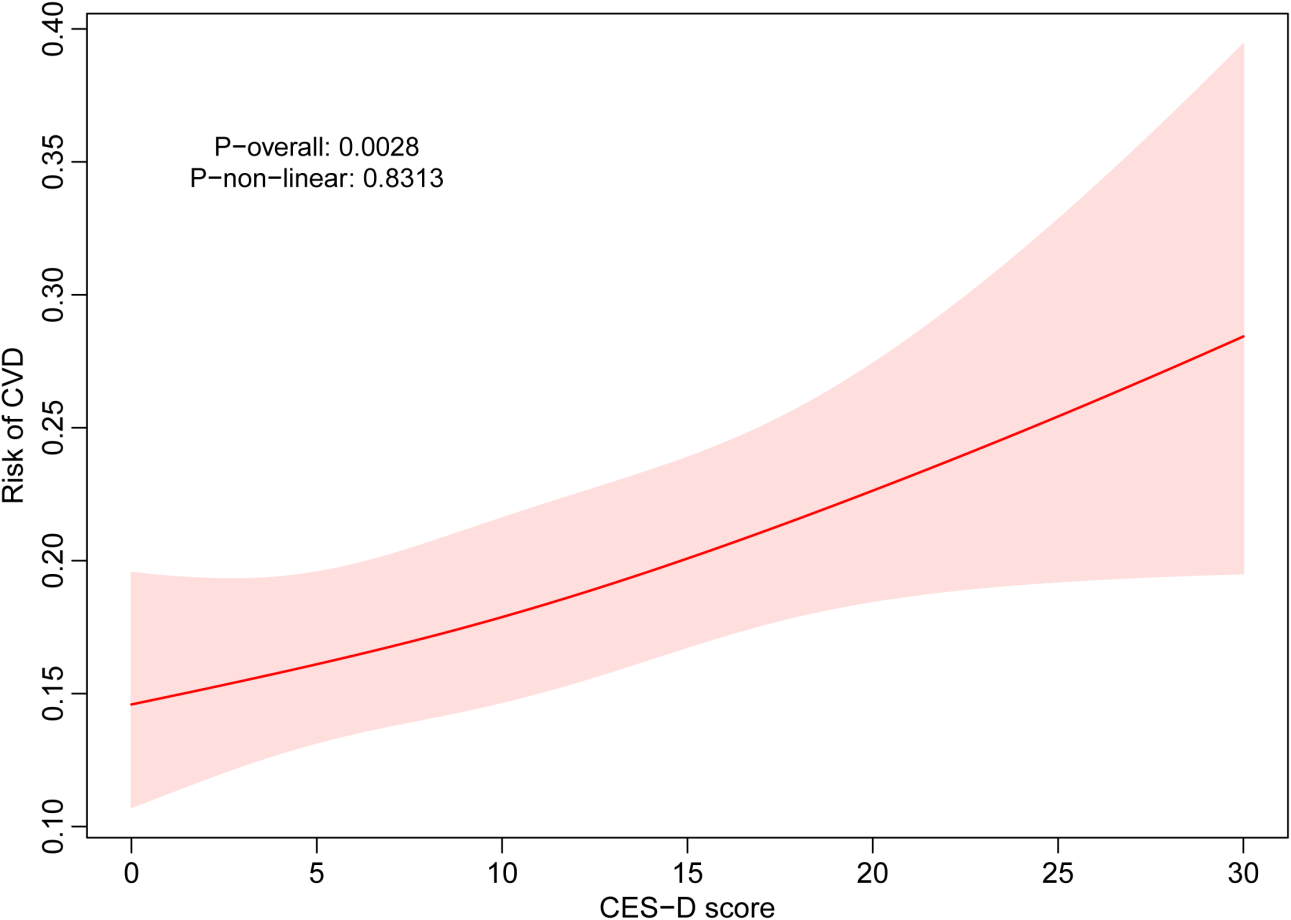
RCS model analysis the nonlinear association between CES-D score and the risk of CVD.

First, we conducted a multivariate logistic regression analysis to investigate the CES-D score (continuous variable) and the risk of CVD (**Table 2**). In all models, a positive correlation was observed between the CES-D score and the risk of CVD, indicating that for every one-point increase in the CES-D score, there was a 2.8% increase in the CVD (OR=1.028, 95% CI = 1.012~ 1.044, P< 0.001). We employed RCS models to investigate the nonlinear and dose-response relationships between depression and CVD. A linear positive association was observed between depression and CVD risk (p non-linear= 0.83, p overall= 0.003), thereby confirming the findings from prior analyses (**Figure 2**). When CES-D score of 12 points was used as the cut-off point to distinguish depressive states, we found that the risk of CVD of depressed individuals (CES-D≥ 12) was 38.8% higher than that of non-depressed individuals (CES-D< 12) (OR=1.388, 95% CI = 1.127~ 1.709, P= 0.002) (**Table 2**).

**Table 2:** Association of depression with the risk of CVD.

Variables	Crude model		Model 1		Model 2	
	OR (95% CI)	p.value	OR (95% CI)	p.value	OR (95% CI)	p.value
Primary analysis						
CES-D (continuous variable)	1.016 (1.002,1.030)	0.027	1.018 (1.003,1.033)	0.016	1.028 (1.012,1.044)	<0.001
Stratified by depression						
Non-depressive (CES-D= 0-11)	ref		ref		ref	
Depressive (CES-D= 12-30)	1.205(1.001,1.450)	0.048	1.222(1.009,1.481)	0.040	1.388(1.127,1.709)	0.002

OR, odds ratios; 95% CI, 95% confidence intervals.

Crude model: no covariates were adjusted; Model 1: sex, age, marital status, educational level and residence were adjusted; Model 2: sex, age, marital status, educational level, residence, smoking status, alcohol status, BMI, Waist circumference, hypertension, DM, TC, HDL, LDL and TG were adjusted.

Crude model: no covariates were adjusted; Model 1: sex, age, marital status, educational level and residence were adjusted; Model 2: sex, age, marital status, educational level, residence, smoking status, alcohol status, BMI, Waist circumference, hypertension, DM, TC, HDL, LDL and TG were adjusted.

To investigate the role of inflammatory status in arthritis patients, participants were stratified into four subgroups based on CRP levels and depression status: (1) low-inflammation subgroup without depression, (2) high-inflammation subgroup without depression, (3) low-inflammation subgroup with depression, and (4) high-inflammation subgroup with depression. Our analysis demonstrated that inflammation levels did not influence cardiovascular event risk in non-depressed patients (OR=1.027, 95% CI = 0.706~ 1.495, P= 0.888), while among depressed patients, the high-inflammation subgroup exhibited significantly elevated cardiovascular event risk compared to the low-inflammation subgroup (OR=1.524, P= 0.024 vs OR=1.324, P= 0.016; P for trend= 0.004) (**Table 3**).

**Table 3:** Synergistic impact of depression and systemic inflammation on CVD.

Group	Crude model		Model 1		Model 2	
	HR (95% CI)	p.value	HR (95% CI)	p.value	HR (95% CI)	p.value
Low crp+ without depression	ref		ref		ref	
High crp+ without depression	1.089 (0.769,1.544)	0.630	1.037 (0.730,1.472)	0.840	1.027 (0.706,1.495)	0.888
Low crp+ with depression	1.172 (0.956,1.438)	0.127	1.144 (0.928,1.411)	0.208	1.324 (1.054,1.665)	0.016
High crp+ with depression	1.493 (1.064,2.094)	0.020	1.46 (1.038,2.053)	0.030	1.524 (1.058,2.196)	0.024
p for trend		0.021		0.039		0.004

OR, odds ratios; 95% CI, 95% confidence intervals.

**Figure 3:**
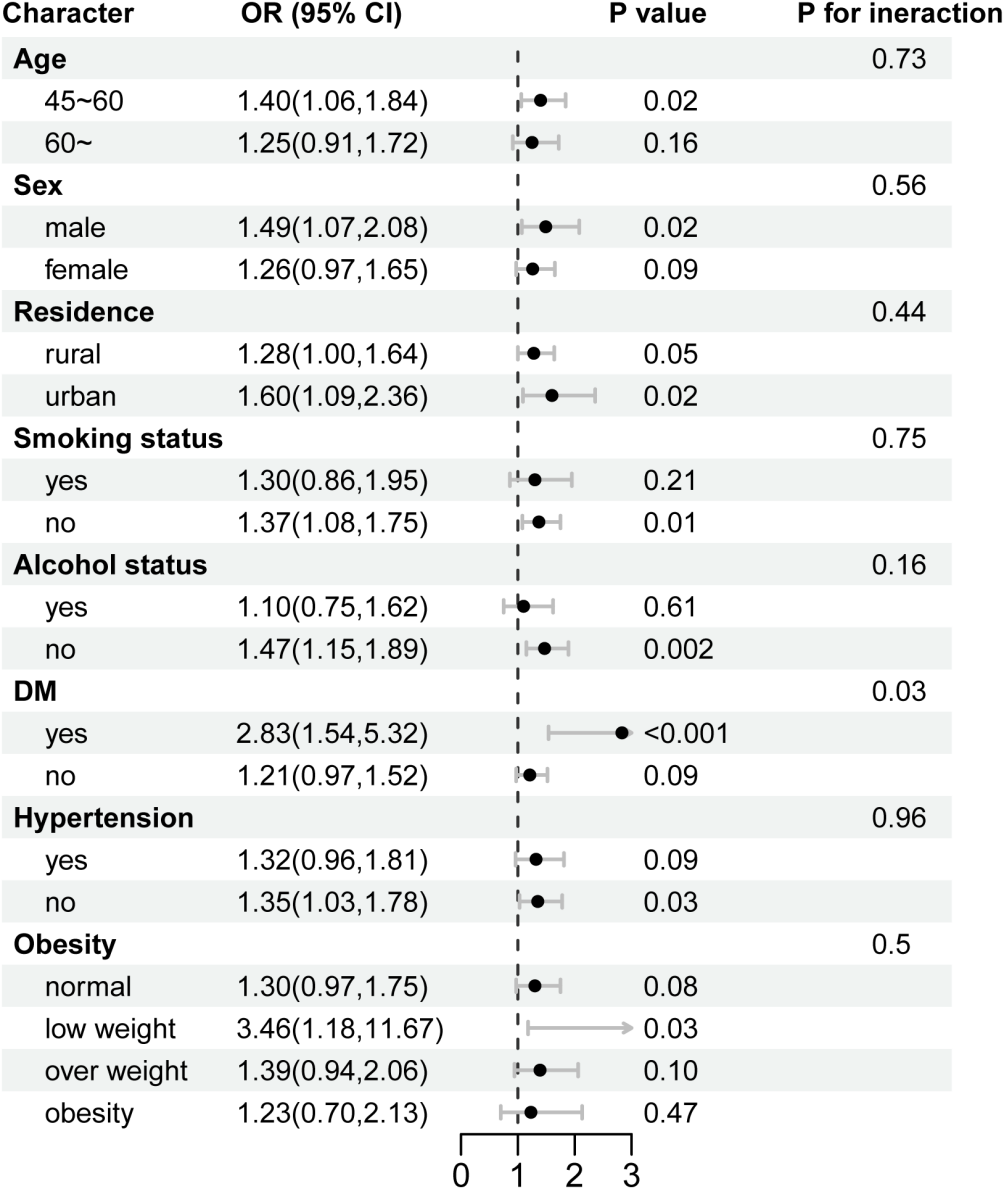
Stratified analysis for the association between depression and CVD. OR, odds ratios; 95% CI, 95% confidence intervals; DM, diabetes mellitus.

**Figure 3** shows the association between depressive states and the risk of CVD stratified by potential risk factors. The association between depressive states and the risk of CVD was more pronounced among participants with DM (OR=2.83, 95% CI = 1.54~ 5.32, P< 0.001), compared with those without DM (OR=1.21, 95% CI = 0.97~ 1.52, P= 0.09) at baseline (P = 0.03 for interaction). Through formal mediation analyses, we quantified the reciprocal pathways between depression and CRP in CVD risk (**Supplementary Figure 1**). The results indicate that the mediating effect was not significant (P= 0.92). This suggests that while both depression and CRP are cardiovascular risk factors, CRP does not function as a primary mediator in this pathway. To validate the robustness of our findings, we conducted sensitivity analyses (**Supplementary Table 3**). First, multiple imputation for missing values showed no significant changes in outcomes. Similarly, complete-case analysis after deleting missing values demonstrated consistent findings across all models.

## 4 Discussion

Our prospective cohort analysis of 2,571 Chinese arthritis patients from the CHARLS database provides compelling evidence for the synergistic interplay between depression and systemic inflammation in elevating CVD risk. The principal findings—a 2.8% increase in CVD odds per CES-D point escalation, amplified risk stratification by CRP levels (38.8-52.4% increased CVD risk in depressed subgroups), and DM status as a critical effect modifier (OR=2.83, P<0.001). The key findings reveal three critical insights: (1) Depression severity, measured by CES-D scores, exhibits a linear dose-response relationship with incident CVD in arthritis patients, independent of traditional metabolic risk factors; (2) Systemic inflammation, quantified by CRP levels, modifies this association, with high inflammation (CRP ≥3 mg/L) amplifying CVD risk specifically in depressed individuals; and (3) DM significantly potentiates the depression-CVD relationship, suggesting shared pathophysiological pathways involving insulin resistance and endothelial dysfunction (30). These results advance our understanding of cardiovascular pathogenesis in arthritis by highlighting depression as a modifiable risk amplifier within the context of chronic inflammation. These findings align with emerging neuro-immunological frameworks while offering novel insights into population-specific risk stratification in aging Asian cohorts—a demographic historically underrepresented in prior research (31).

There were no causes of unnatural death among these depressed arthritis patients, with the most common causes of death being cardiovascular diseases and strokes (32). Depression can significantly increase CVD risk in patients with arthritis through several mechanisms. Firstly, both conditions involve chronic inflammation, with elevated pro-inflammatory cytokines like IL-6 and TNF-α exacerbating arthritis symptoms and contributing to CVD, a major cause of death in arthritis patients (33). Depression is associated with dysregulation of the autonomic nervous system, including reduced heart rate variability and increased sympathetic nervous system activity, which are a significant cause of increased CVD in arthritis patients (34). Secondly, depression can negatively impact health behaviors, leading to poor disease management in arthritis patients. Depressed individuals are more likely to have poor medication adherence, reduced physical activity, unhealthy diet, and increased smoking or alcohol use (35).

The bidirectional relationship between depression and inflammation has been increasingly recognized as a driver of vascular dysfunction (36, 37). Our observation that CRP ≥3 mg/L amplified CVD risk exclusively in depressed patients (OR=1.524 vs. 1.324 in low-inflammation counterparts) suggests that depression may prime the vascular endothelium to inflammatory insults. This aligns with preclinical evidence showing that chronic stress upregulates IL-6 and TNF-α production via hypothalamic-pituitary-adrenal (HPA) axis dysregulation, thereby exacerbating endothelial oxidative stress and plaque instability (6). Recent studies further suggest that peripheral inflammatory cytokines may directly alter blood-brain barrier permeability, enabling microglial activation and central nervous system inflammation that perpetuates depressive symptoms (38). Notably, the lack of association between CRP and CVD in non-depressed individuals implies that inflammation alone is insufficient to trigger cardiovascular events without concurrent psychological comorbidity.

Furthermore, our stratified analysis revealed a striking 2.83-fold CVD risk (95% CI: 1.54–5.32) among depressed arthritis patients with DM. Notably, the DM-depression interaction in CVD risk amplification suggests insulin resistance as a shared mediator. Preclinical models demonstrate that chronic stress and hypercortisolism in depression impair insulin signaling through glucocorticoid receptor-mediated suppression of IRS-1 phosphorylation (39). Concurrently, inflammatory cytokines like IL-1β and TNF-α induce serine phosphorylation of insulin receptor substrates, establishing a molecular bridge between inflammation, depression, and metabolic dysfunction (40). These findings underscore the necessity of multidisciplinary care models addressing metabolic, inflammatory, and psychiatric comorbidities in high-risk populations.

The differential CVD risk observed across inflammatory strata carries immediate clinical implications. Current arthritis management guidelines prioritize traditional CVD risk factors but inadequately address psychosocial comorbidities (41). Our data advocate for integrated care models incorporating depression screening and CRP monitoring in arthritis patients, particularly those with diabetes. Meanwhile, emerging evidence suggests that depression and arthritis share common therapeutic targets, particularly within neuroimmune pathways and inflammatory signaling cascades (42); our findings provide empirical support for this paradigm shift.

While our study benefits from robust epidemiological methods—including RCS modeling of dose-response relationships and comprehensive sensitivity analyses—several limitations warrant discussion. First, CRP measurement at a single time point may not fully capture chronic inflammatory burden. Substantial intraindividual variability in inflammatory biomarkers, potentially attenuating observed effect sizes. Second, residual confounding from unmeasured factors like dietary patterns, physical activity, or genetic predisposition (e.g., shared HLA alleles between depression and autoimmune diseases) cannot be excluded. Third, the CHARLS cohort’s reliance on self-reported CVD diagnoses, though validated in prior studies (22), may underestimate asymptomatic cardiovascular pathology. Fourth, the assessment of systemic inflammation relied solely on CRP. While CRP is a well-validated, widely used biomarker for systemic inflammation and is strongly associated with cardiovascular risk, it does not capture the full spectrum of inflammatory activity, particularly in RA. Comprehensive assessment of inflammatory status in RA typically incorporates composite clinical measures (such as DAS28, SDAI, CDAI), patient-reported outcomes (e.g., VAS pain/global assessment), and serological markers (e.g., RF, ACPA) alongside CRP/ESR. Regrettably, the CHARLS database, as a large-scale, population-based cohort study primarily focused on aging, health, and retirement, does not include these RA-specific clinical measures, detailed patient-reported outcome scales specific to arthritis activity, or serological markers like RF and ACPA. Consequently, our findings on the modifying role of “inflammatory status” are based solely on systemic CRP levels and may not fully reflect the joint-specific inflammatory burden or disease activity state in participants with RA within the cohort. This limitation underscores the need for future studies in dedicated RA cohorts with comprehensive inflammatory phenotyping to confirm and extend our observations regarding the interplay between depression, inflammation, and CVD risk.

This large-scale prospective study establishes depression as an independent cardiovascular risk amplifier in arthritis patients, with systemic inflammation serving as a critical effect modifier. The synergistic risk observed in high-inflammation depressed individuals underscores the need for combinatorial therapeutic strategies targeting both psychological distress and immuno-metabolic dysregulation. As global arthritis prevalence rises alongside urbanization-associated stressors, our findings advocate for redefining cardiovascular prevention paradigms to incorporate mental health and inflammatory monitoring. However, future longitudinal studies of higher methodological rigor are warranted to elucidate the causal relationship between the depression and CVD.

## Data Availability

The original contributions presented in the study are included in the article/**Supplementary Material**. Further inquiries can be directed to the corresponding author.
