# Supplementary material for: Association between the depression and cardiovascular risk in arthritis patients: a prospective cohort study from the CHARLS database
Source: Front Immunol. 2025 Aug 19;16:1590483. doi: 10.3389/fimmu.2025.1590483 (PMC12401986; doi:10.3389/fimmu.2025.1590483)
Supplement: Supplementary file 1 [file DataSheet1.docx]

Supplementary Table 1 The VIF values for all variables

|  |  |
| --- | --- |
| **Variable** | **VIF values** |
| Age | 1.96 |
| Sex | 1.32 |
| Marital status | 1.12 |
| Education level | 1.91 |
| Residence place | 1.07 |
| Smoking | 1.69 |
| Drinking state | 1.29 |
| BMI | 1.84 |
| WC | 1.65 |
| Hypertension | 1.13 |
| DM | 1.08 |
| Total cholesterol | 8.49 |
| Triglycerides | 5.93 |
| Low-density lipoprotein cholesterol | 8.50 |
| High-density lipoprotein cholesterol | 3.25 |

Supplementary Table 2 Baseline characteristics of participants excluded and include in the study

| **Variable** | **Total (n=17708)** | **Exclude (n=15137)** | **Include (n=2571)** | **p.value** |
| --- | --- | --- | --- | --- |
| Age (yr), Mean (SE) | 58.99 ± 10.16 | 59.02 ± 10.41 | 58.72 ± 8.51 | 0.31 |
| Sex |  |  |  | **<0.0001** |
| Female n (%) | 9221(52.12) | 7706(50.90) | 1515(59.00) |  |
| Male n (%) | 8471(47.88) | 7418(49.00) | 1053(41.00) |  |
| Marital status |  |  |  | **<0.01** |
| Married n (%) | 15417(87.22) | 13125(86.70) | 2292(89.15) |  |
| Other n (%) | 2258(12.78) | 1979(13.07) | 279(10.85) |  |
| Educational level |  |  |  | **<0.0001** |
| Middle school or above n (%) | 5898(33.38) | 5284(34.91) | 614(23.90) |  |
| No formal education n (%) | 7859(44.52) | 6501(42.95) | 1358(52.86) |  |
| Primary school n (%) | 3896(22.07) | 3299(21.79) | 597(23.24) |  |
| Residence |  |  |  | **<0.0001** |
| Rural n (%) | 10537(59.51) | 8727(57.65) | 1810(70.40) |  |
| Urban n (%) | 7168(40.49) | 6407(42.33) | 761(29.60) |  |
| BMI (kg/m2), Mean (SE) | 23.47 ± 3.89 | 23.45 ± 3.89 | 23.56 ± 3.94 | 0.19 |
| WC (cm), Mean (SE) | 84.28 ± 12.62 | 84.35 ± 12.60 | 83.90 ± 12.64 | 0.15 |
| CRP (mg.L), Mean (SE) | 2.80 ± 7.63 | 2.90 ± 8.07 | 2.48 ± 5.92 | **<0.01** |
| TC (mg.dL), Mean (SE) | 192.97 ± 38.89 | 192.20 ± 38.71 | 195.45 ± 39.55 | **<0.0001** |
| HDL-C (mg.dL), Mean (SE) | 50.84 ± 15.33 | 50.47 ± 15.28 | 52.15 ± 15.45 | **<0.0001** |
| LDL-C (mg.dL), Mean (SE) | 115.99 ± 34.91 | 115.77 ± 34.90 | 116.65 ± 35.04 | 0.19 |
| TG (mg.dL), Mean (SE) | 134.91 ± 110.26 | 134.15 ± 105.94 | 137.11 ± 124.00 | 0.20 |
| Smoking status |  |  |  | 0.54 |
| No n (%) | 12695(72.31) | 10828(71.53) | 1867(72.62) |  |
| Yes n (%) | 4862(27.69) | 4158(27.47) | 704(27.38) |  |
| Alcohol status |  |  |  | 0.45 |
| No n (%) | 11783(67.14) | 10045(66.36) | 1738(67.60) |  |
| Yes n (%) | 5767(32.86) | 4934(32.60) | 833(32.40) |  |
| DM |  |  |  | 0.08 |
| No n (%) | 15481(88.12) | 13239(87.46) | 2242(87.20) |  |
| Yes n (%) | 2087(11.88) | 1758(11.61) | 329(12.80) |  |
| Hypertension |  |  |  | 0.05 |
| No n (%) | 10960(62.41) | 9308(61.49) | 1652(64.28) |  |
| Yes n (%) | 6600(37.59) | 5682(37.54) | 918(35.72) |  |

yr= year, SE= standard error, BMI= body mass index, WC= waist circumference, CRP= C-reactive protein, TC= total cholesterol, HDL-C= high-density lipoprotein cholesterol, LDL-C= low-density lipoprotein cholesterol, TG= triglycerides, DM= diabetes mellitus, CVD= cardiovascular disease; All the above mean, standard error, and percentage of frequency were weighted except for the frequency.

Supplementary Table 3. Association of depression with the risk of CVD before and after sensitivity analyses

|  | Complete case | | Multiple imputation | | Excluding missing data | |
| --- | --- | --- | --- | --- | --- | --- |
|  | OR | p.value | OR | p.value | OR | p.value |
| Primary analysis |  |  |  |  |  |  |
| CES-D (continuous variable) | 1.028 | <0.001 | 1.024 | 0.002 | 1.028 | <0.001 |
| Stratified by depression |  |  |  |  |  |  |
| Non-depressive (CES-D= 0-11) | ref |  | ref |  | ref |  |
| Depressive (CES-D= 12-30) | 1.388 | 0.002 | 1.31 | 0.007 | 1.388 | 0.002 |

Sex, Age, Marital status, Educational level, Residence, Smoking status, Alcohol status, BMI, Waist circumference, Hypertension, DM, TC, HDL, LDL and TG were adjusted.


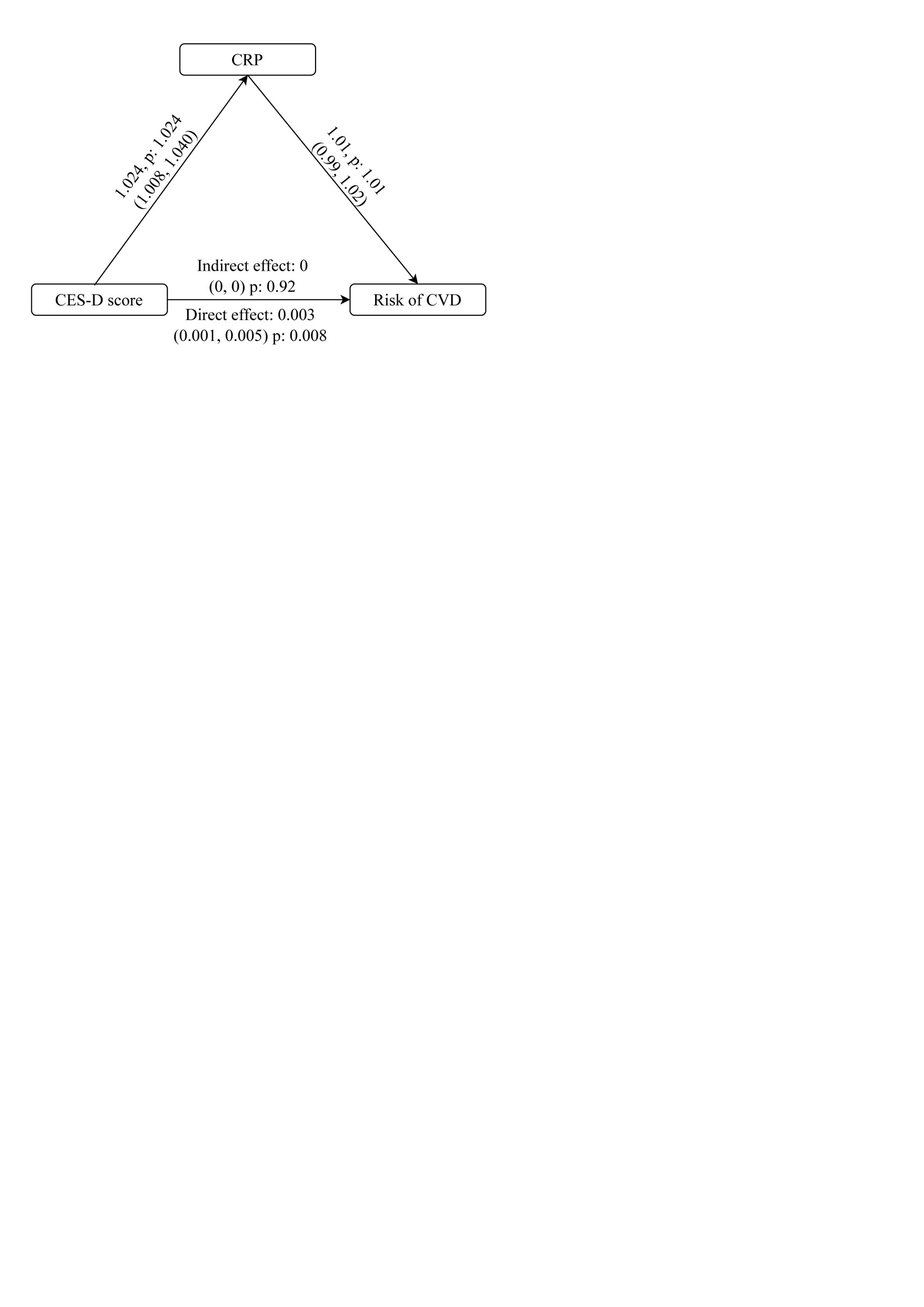


Supplementary Figure 1. Mediation analyses of depression and CRP on the risk of CVD
